# Y-shaped DNA as a dynamic self-assembly nanomaterial for phenotype-specific regulation of stem cell differentiation on the gene level

**DOI:** 10.1093/rb/rbaf043

**Published:** 2025-05-14

**Authors:** Wengang Liu, Ruili Liu, Lok Ting Chu, Xinlei Wang, Jianpeng Wu, Jiandong Ding, Ting Hsuan Chen

**Affiliations:** Department of Biomedical Engineering, City University of Hong Kong, Hong Kong Special Administrative Region 999077, China; State Key Laboratory of Molecular Engineering of Polymers, Department of Macromolecular Science, Fudan University, Shanghai 200438, China; Department of Biochemistry and Molecular Biology, Guang Dong Medical University, Zhanjiang 524023, China; State Key Laboratory of Molecular Engineering of Polymers, Department of Macromolecular Science, Fudan University, Shanghai 200438, China; Department of Biomedical Engineering, City University of Hong Kong, Hong Kong Special Administrative Region 999077, China; State Key Laboratory of Molecular Engineering of Polymers, Department of Macromolecular Science, Fudan University, Shanghai 200438, China; Department of Biomedical Engineering, City University of Hong Kong, Hong Kong Special Administrative Region 999077, China; City University of Hong Kong Shenzhen Research Institute, Shenzhen 518057, China; Hong Kong Centre for Cerebro-Cardiovascular Health Engineering, Hong Kong Science Park, Hong Kong Special Administrative Region 999077, China

**Keywords:** mesenchymal stem cell, Y-shaped DNA, osteogenic differentiation, micropattern, poly(ethylene glycol), antisense oligonucleotide, gene regulation

## Abstract

While genetic engineering has offered new strategies for regulating stem cell differentiation, the efficacy varies in cells with different phenotypes or lineage commitments, leading to inconsistent differentiation outcomes and uncertainty in regenerative medicine. To address this issue, we employ a Y-shaped DNA (Y-DNA) as a nanomaterial to phenotype-specifically regulate differentiation of human mesenchymal stem cells (hMSCs). Y-DNA is composed of three DNA strands with complementary sequences and different roles. The Y-DNA designed in the present study can be uniquely activated by miR-106a-5p, a microRNA preferentially expressed in adipogenesis-biased hMSCs. Upon activation, the Y-DNA disassembles, releasing an antisense oligonucleotide that inhibits expression of cofilin, which serves as a key regulator to enhance adipogenic differentiation, and thus, prevents hMSCs from undergoing osteogenic differentiation. The key regulatory role of cofilin in hMSC differentiation is verified at the single-cell level on arginine–glycine–aspartate microislands under the nonfouling background of poly(ethylene glycol) hydrogels. Our strategy effectively redirects these cells towards osteogenic differentiation by inhibiting adipogenic differentiation, demonstrating dose dependence with high specificity, selectivity, and low toxicity. hMSCs cultured in a dual induction medium (a mixture of adipogenic medium and osteogenic medium) show enhanced osteogenic differentiation after transfection with the nanostructured Y-DNA. This approach addresses the challenge of cell heterogeneity in bone regeneration, offering a promising solution for precise control over stem cell fate. The ability of Y-DNA to specifically target cells with a propensity for adipogenic differentiation and to reprogram their lineage commitment has significant implications for the field of regenerative medicine, particularly in applications requiring enhanced purity of cell differentiation outcomes.

## Graphical Abstract

**Figure:**
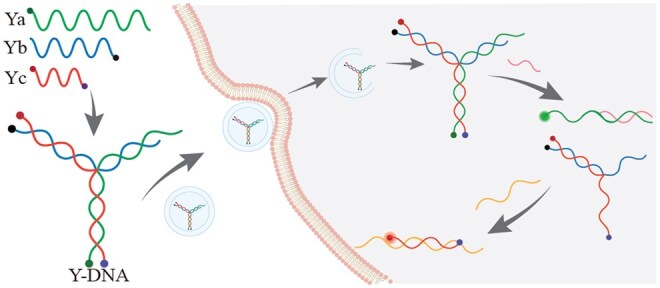

## Introduction

Human mesenchymal stem cells (hMSCs) are essential in regulating bone homeostasis [1] because of their capacity of differentiating into osteoblasts [2, 3]. Consequently, hMSCs have been extensively utilized for bone repair, either when being combined with a scaffold or directly injected into the damaged area [4–8]. It is known that the osteogenic differentiation of mesenchymal stem cells (MSCs) competes with other linage commitment of stem cells, in particular, adipogenesis [9–11]. Therefore, it is highly desired to develop an approach to up-regulate osteogenic differentiation of hMSCs while simultaneously downregulating other competing lineage commitment.

Genetic engineering has provided great potential to guide cell differentiation [12–15]. Various methods have been developed to enhance the osteogenic potential of MSCs such as gene activation using CRISPR, inhibition or overexpression of microRNAs (miRNAs), and the introduction of osteogenesis-related genes using gene gun [16–20]. Among them, antisense oligonucleotide (ASO) as a single-stranded nucleotide can bind to the target mRNA, leading to mRNA degradation and eventually translational inhibition [21]. This mechanism has been used in gene therapy with new drug approvals [22, 23]. To modulate the differentiation of MSCs, for instance, the knockout of lnc13728 [24] or LYPLAL1-antisense RNA1 [25] can effectively reduce the adipogenic differentiation. Similarly, the inhibition of let-7 miRNA [26] can enhance the osteogenic differentiation potential. Furthermore, the suppression of Sirtuin-1 [27] has been shown to regulate chondrogenic differentiation. Despite these achievements, the use of ASO still face challenges [21]. First, single-stranded ASOs often exhibit off-target effects that lead to cytotoxicity [28, 29]. Second, the intrinsic heterogeneity within the same batch of cells may result in the uncertainty in tissue regeneration [30]. With such heterogeneity, the efficacy of those genetic engineering may vary for cells with mixed phenotypes or during lineage commitment. Thus, an ideal ASO should be activated within a specific cell type, thereby enabling targeted gene silencing functionality.

**Figure 1.**
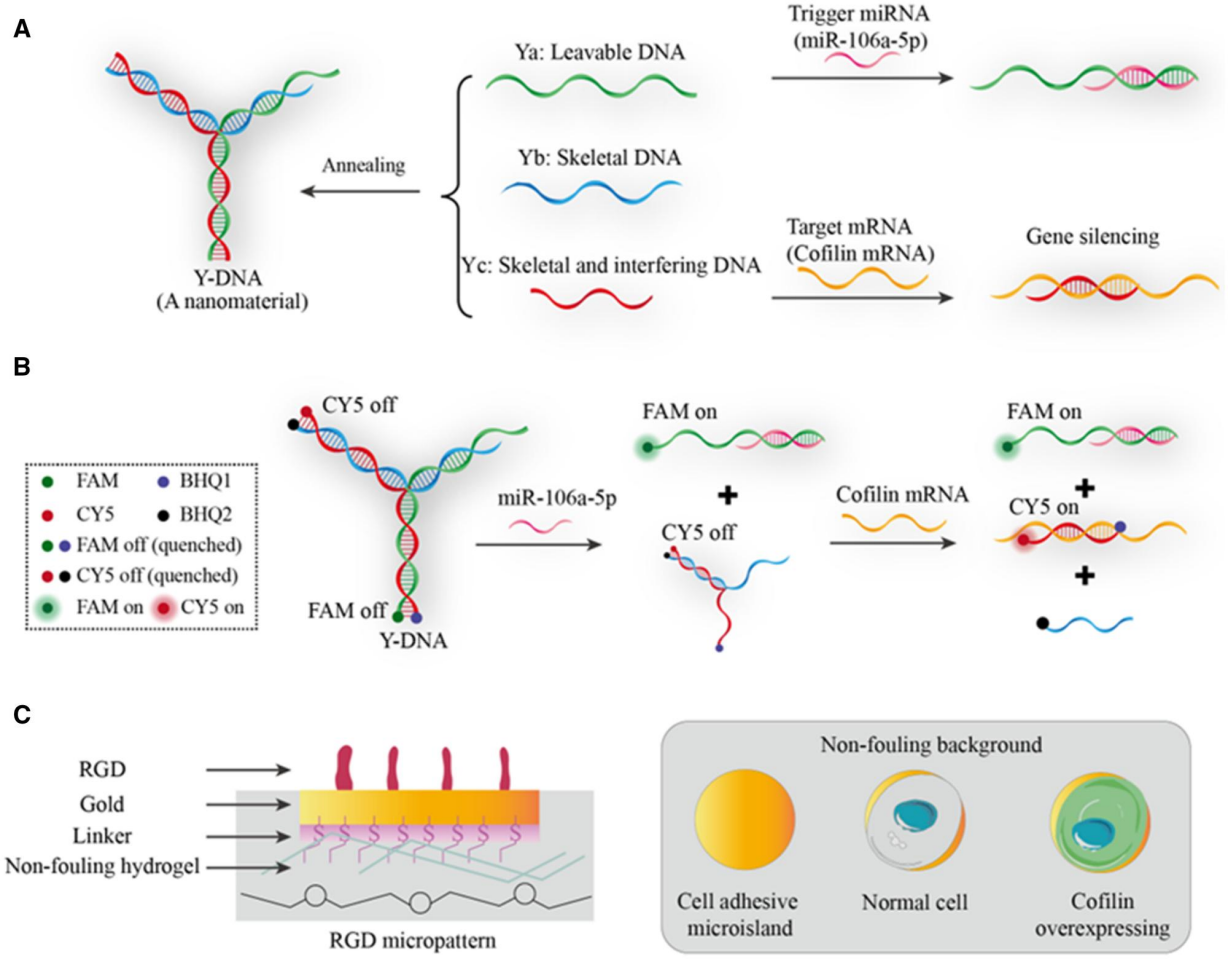
Schematic of the present study. (**A**) The formation of Y-DNA and the functions of each single strands. Three single strands, Ya, Yb and Yc, anneal to form Y-DNA. A portion of the bases in Ya can be complementary to the target miRNA, leading to the disassembly of Y-DNA. Yb serves as a skeletal DNA, while Yc contains an ASO sequence that binds to the target mRNA for gene silencing. (**B**) Fluorescence technique to reflect the miRNA activation of Y-DNA for selective gene silencing. To illustrate the mechanism of Y-DNA, the 5' end of Ya is modified with a FAM fluorophore, and the complementary region in Yc carries the quenching group BHQ1. Conversely, the 5' end of Yc is modified with the CY5 fluorophore, which pairs with the quenching group BHQ2 in Yb. Upon encountering miR-106a-5p, Y-DNA disassembles, resulting in green fluorescence. Subsequently, if Yc binds to cofilin mRNA, red fluorescence is emitted. (**C**) Schematic side view (left) and top view (right) of an arginine-glycine-aspartate (RGD) micropattern on the non-fouling poly(ethylene glycol) (PEG) hydrogel to achieve single-cell location of adhesive microislands with an appropriate size and then to examine differentiation fraction for those cells overexpressing cofilin, a key regulator of hMSC differentiation.

Previous studies have revealed the crucial regulatory role of cofilin in the differentiation direction of MSCs [31–33]. Its overexpression enhances the potential of adipogenesis, while inhibiting the osteogenic potential [31, 32]. Here, we designed a Y-shaped DNA (Y-DNA, Figure 1A and B) to achieve phenotype-specific activation of the ASO targeting cofilin. The DNA nanostructure is formed by self-assembly of three single-stranded DNA (ssDNA). In the absence of the target microRNA miR-106a-5p, the structure remains intact, preventing the non-specific release of ASO. In contrast, for cells with high expression of miR-106a-5p, which is associated with early adipogenic differentiation [34–36], the miR-106a-5p disassembles the Y-DNA, allowing the preferential release of ASO in cells biased towards adipogenic differentiation. The ASO inhibits cofilin expression, causing a decrease of adipogenic differentiation, and thus, an increase in the osteogenic potential of these cells. Together, using the Y-shaped DNA as a dynamic nanomaterial to inhibit cofilin expression on the transcriptional level, we attempt to upregulate osteogenic differentiation of hMSCs through downregulating adipogenic commitment.

## Materials and methods

### Fabrication of micropatterned surfaces

The fabrication of gold microislands on PEG hydrogels was performed following our previous protocol [37]. Briefly, photolithography was used to prepare micropatterned glass. Glass slides were cleaned by piranha solution (H_2_SO_4_: H_2_O_2_ = 3:1) at 110°C for 1 hr. After drying, hexamethyldisilazane (Sigma-Aldrich) was coated on the surface of slides. AZ5214 photoresist (AZ Electronic Materials) was then spin-coated on the slides at 3000 rpm for 30 s and baked at 95°C for 2 mins. After UV exposure and development, the slides were dried under compressed air. Gold was sputtered on these slides and then rinsed with acetone in an ultrasonic cleaning machine to get gold micropatterns. Before grafting the linker, the slides were treated with a 2-trimethoxysilane (Gelest) solution (6 mM in toluene) at 60°C for 24 hr. Next, the slides were washed with toluene in an ultrasonic cleaning machine. Then, the glass slides were put into an ethanol solution of 1 mM N, N′-bis(acryloyl) cystamine (Alfa Aesar) at 35°C for 1 hr to graft the linker on the gold microislands and rinsed the excess linkers with ethanol. After that, a mixture of poly(ethylene glycol) diacrylate (PEGDA) (Sigma-Aldrich, MW 700) and the initiator 2-hydroxy-40-(2-hydroxyethoxy)-2-methylpropiophenone (Sigma-Aldrich) was cast on the surface of the glass slide. After curing by UV light for 60 min, the PEG hydrogel was peeled off and the gold microislands were transferred to the surface of the hydrogel. Prior to seeding cells on the PEG hydrogel, the gold microislands were modified with thiol-end RGD (Synpeptide Co., Ltd) by S-Au bond so that cells can adhere to them.

### Preparation of Y-DNA

The designed ssDNA and mimics (Supplementary Table S1) were synthesized by Sangon Biotech (Shanghai) Co., Ltd. To prevent DNA degradation by exonucleases in cells and reduce the attack of endonucleases, the entire DNA strand was modified with phosphorothioate [38]. Y-DNA was constructed by mixing the three strands Ya, Yb and Yc at the same concentration with buffer (100 mM Tris-HCl, 1 mM CaCl_2_, and 25 mM MgCl_2_, pH 7.0). The mixture was then annealed at 95°C for 5 min and gradually cooled to 25°C at a rate of 0.5°C/min. We also prepared two models of Y-shape DNAs, serving as controls to confirm the dynamic properties of the Y-DNA nanostructure. The formation of the two models used the same method as the experimental Y-DNA, using the corresponding ssDNA (Supplementary Table S1). The final concentrations of Y-shaped DNAs and mimics in all reaction conditions were 400 nM and 600 nM, respectively, and the reaction time for each step was 1 hr, unless otherwise stated.

### Characterization of Y-DNA

Native polyacrylamide gel electrophoresis (PAGE, Biosharp) was used to confirm the successful formation of Y-DNA. The annealing products were mixed with loading buffer (TaKaRa), and then, loaded into a 4 to 15% native PAGE gel. They were run in 1× tris-borate-ethylenediaminetetraacetic acid (TBE) buffer (Promega) at 80 V for 30 min, and then, at 100 V for another 50 min. The gel was stained with 1× GelRed (Biotium) for 15 min after the run, rinsed three times with 1× TBE buffer, and visualized using BioRad ChemiDoc MP Imaging System.

To test the binding ability of Y-DNA and the mimic of miR-106a-5p T_miR106_, Y-DNA with a concentration of 400 nM was reacted with T_miR106_ at concentrations ranging from 5 to 600 nM at 37°C for 1 hr, or with T_CFL_ at concentrations ranging from 5 to 600 nM at 37°C for 1 hr after reacting with 600 nM T_miR106_ (fully activated Y-DNA). For selective experiments, miRNA mimics such as T_miR27_, T_miR124_, T_miR29_ and T_miR221_ (Supplementary Table S1) were used. At the same time, alkaline phosphatase (ALP) mRNA mimic with T_miR106_ to represent the specific release of ASO. After reaction, the signals of FAM with excitation wavelength (ex) 490 nm and emission wavelength (em) 510–750 nm and CY5 (ex: 646 nm; em: 655–750 nm) of the reactants were measured using a HORIBA FluoroMax-4 fluorescence spectrometer.

### Cell culture and transfection

Undifferentiated hMSCs (Lonza) were cultured in low-glucose Dulbecco’s modified Eagle medium (DMEM, Gibco) supplemented with 10% fetal bovine serum (FBS, Gibco) and 1% penicillin/streptomycin (P/S, Gibco). Lonza’s adipogenic induction medium (AM) and osteogenic induction medium (OM) were used for cell induction. The cells were seeded in cell culture dishes at a density of 1.2 × 10^4^ cells/cm^2^ and incubated at 37°C in a humidified atmosphere of 5% CO_2_ and 95% air. All experiments used cells from early passages (up to passage 8).

For cofilin overexpression, cells were transfected with plasmid (a gift from James Bamburg, Addgene plasmid # 50859 [39]) via electroporation according to the Lonza’s protocol. Briefly, 5 × 10^4^ cells were washed with PBS and resuspended in a mixture of plasmid and 20 µl of P1 primary cell 4D-Nucleofector™ X kit (16.4 µl of Nucleofector™ solution and 3.6 µl of supplement). The cell mixture was then subjected to the FF-104 program in the Lonza 4D-Nucleofector system. After transfection, cells were plated in cell culture microplate with growth medium for further experiments.

The DNA nanostructure was transfected by Lipofectamine™ stem transfection reagent (Invitrogen) after induction with dual induction medium (mixing equal volumes of adipogenic and osteogenic media) for 3 days. In brief, the transfection reagent and DNA were separately diluted in Opti-MEM™ I reduced serum medium (Gibco) in equal volumes and incubated for 10 min. Subsequently, the DNA-lipid complex was added to the cells and incubated for 2 days, followed by 7 days of culture using dual induction medium before RNA extraction or oil red O and fast blue RR staining.

### Evaluation of cell differentiation

ALP and lipid droplets were used as indicators of osteoblasts and adipocytes, respectively. The staining procedure followed the manufacturer's instructions. First, cells were fixed with citrate solution and rinsed with deionized (DI) water. Then, cells were incubated in fast blue RR (Sigma-Aldrich) mixture at room temperature for 30 min. Next, cells were washed with DI water and treated with 60% isopropanol (IPA, Anaqua) for 5 min before being stained with oil red O (Sigma-Aldrich) in IPA for 15 min. After that, PBS was used to wash cells. Color images were taken with the Nikon Eclipse Ti-S microscope. The ALP activity was quantitated using the microplate reader (Molecular Devices SpectraMax M5e) to measure optical density (OD) at wavelength of 570 nm. For cells cultured on micropatterns, the cell differentiation was evaluated by counting the percentages of osteoblast/adipocytes, while undifferentiated hMSCs were excluded.

### Cytotoxicity analysis

CellTiter 96^®^ Aqueous One Solution cell proliferation assay (MTS, Promega) was employed to assess cytotoxicity, following the manufacturer’s protocol. First, cells were induced with the corresponding induction medium for 3 days prior to transfection. After transfection 2 days, cells were incubated with Opti-MEM™ I reduced serum medium containing MTS solution (medium: MTS = 5:1) for 1 hr in a humidified, 5% CO_2_ atmosphere. Following incubation, the absorbance was measured at 490 nm using a microplate reader.

### Quantitative polymerase chain reaction

Total RNAs were extracted using the PureLink RNA mini kit (Invitrogen), and their concentrations were measured using a spectrophotometer (BioDrop μLite). RNA solutions were mixed with the iScript™ cDNA synthesis kit (BioRad) to convert them into cDNA. The procedure and thermal cycles were conducted following the manufacturer’s instructions. The synthesized cDNA was added to SsoAdvanced universal SYBR Green Supermix (BioRad), together with forward and reverse primers designed following the manufacturer’s instructions. The primers (Supplementary Table S2) used are for peroxisome proliferator-activated receptor gamma (PPARγ), ALP and cofilin 1, while the glyceraldehyde 3-phosphate dehydrogenase (GAPDH) primer would act as the endogenous normalizer. The sealed strips were loaded into the real-time PCR system (BioRad) in a thermal cycler over polymerase activation and DNA denaturation at 95°C for 45 s. Under the 44 amplification cycles with denaturation at 95°C for 15 s and annealing with extension at 60°C for 1 min, a melt-curve was obtained. All samples were repeated three times. The quantitative polymerase chain reaction (qPCR) results were analyzed by relative expression of fold changes by ΔΔCt (cycle threshold method). The fold change, which reveals the difference in expression, was calculated based on the equation 2^−ΔΔCt^. In this equation, ΔΔCt means ΔCt of transfected cells minus ΔCt of control, and ΔCt represents the threshold cycle of target gene by GAPDH.

We extracted miRNAs using miRcute miRNA isolation kit (Tiangen). cDNA was synthesized by miRcute plus miRNA first-strand cDNA kit (Tiangen), and then the qPCR experiment was performed using miRcute plus miRNA qPCR kit (SYBR Green, Tiangen). The primer used was Has-106 (Tiangen), while the U6 primer (Tiangen) would act as the endogenous normalizer. All procedures and thermal cycles were conducted following the manufacturer’s instructions. Briefly, the sealed strips were loaded into the real-time PCR system (BioRad) in a thermal cycler over polymerase activation and DNA denaturation at 95°C for 15 min. Under the 44 amplification cycles with denaturation at 95°C for 20 s and annealing with extension at 60°C for 34 s, a melt-curve was obtained. All samples were repeated three times. Data analysis was performed using the methods mentioned above.

### Western blot analysis

The cell pellets were lysed on ice with radio immunoprecipitation assay (RIPA) buffer from Cell Signaling Technology (CST) supplemented with protease inhibitor cocktail also from CST for 45 min. The lysate was then centrifuged at 14 000 g at 4°C for 10 min, and the supernatant was collected as the extracted protein. The Pierce™ BCA protein assay kit (Thermo Scientific) was used to quantify protein concentration according to the manufacturer’s instructions. Fifteen microgram protein lysate was dissolved in NuPAGE™ LDS sample buffer (Invitrogen) supplemented with NuPAGE™ sample reducing agent (Invitrogen) and denaturing electrophoresis was run at 70°C for 10 min. Proteins were first separated by 4–12% tris-glycine PAGE gel (Beyotime) electrophoresis and transferred to a 0.45 μm pore size poly(vinylidene difluoride) (PVDF) membrane (Thermo Scientific). The membrane was blocked for an hour at room temperature (RT) by Western blocking buffer (Beyotime) before incubating in primary antibody under 4°C overnight with following dilution: GAPDH (1:1000 dilution, CST), cofilin (1:800 dilution, Santa Cruz). After being washed in the Western wash buffer (Beyotime), the membrane was incubated with horseradish peroxidase (HRP) conjugated secondary antibody (1:2000 dilution, CST) for an hour at RT. The protein expression was detected using enhanced chemiluminescence (ECL) like Western reagent (Beyotime) and the Bio-rad ChemiDoc MP Imaging System.

## Results

### Fabrication of the micropatterned surface to achieve single cell adhesion

Here, we prepared circular gold microislands with cell-adhesive RGD decoration on nonfouling hydrogels (Figure 1C). The pattern is fabricated by photolithography on glass slides and subsequently transferred to PEG hydrogels [40–43] providing a unique platform to investigate cell adhesion, proliferation, migration and differentiation, which are known to be influenced by the physical and chemical properties of the material surfaces [44–49]. Importantly, because of the strongly fouling-resistant background of PEG hydrogels, it allows tracing differentiation fate of a specific cell. By selecting an appropriate size of microislands [50], a single-cell adhesion can be achieved.

### Validation of the target gene cofilin and the trigger miRNA

**Figure 2.**
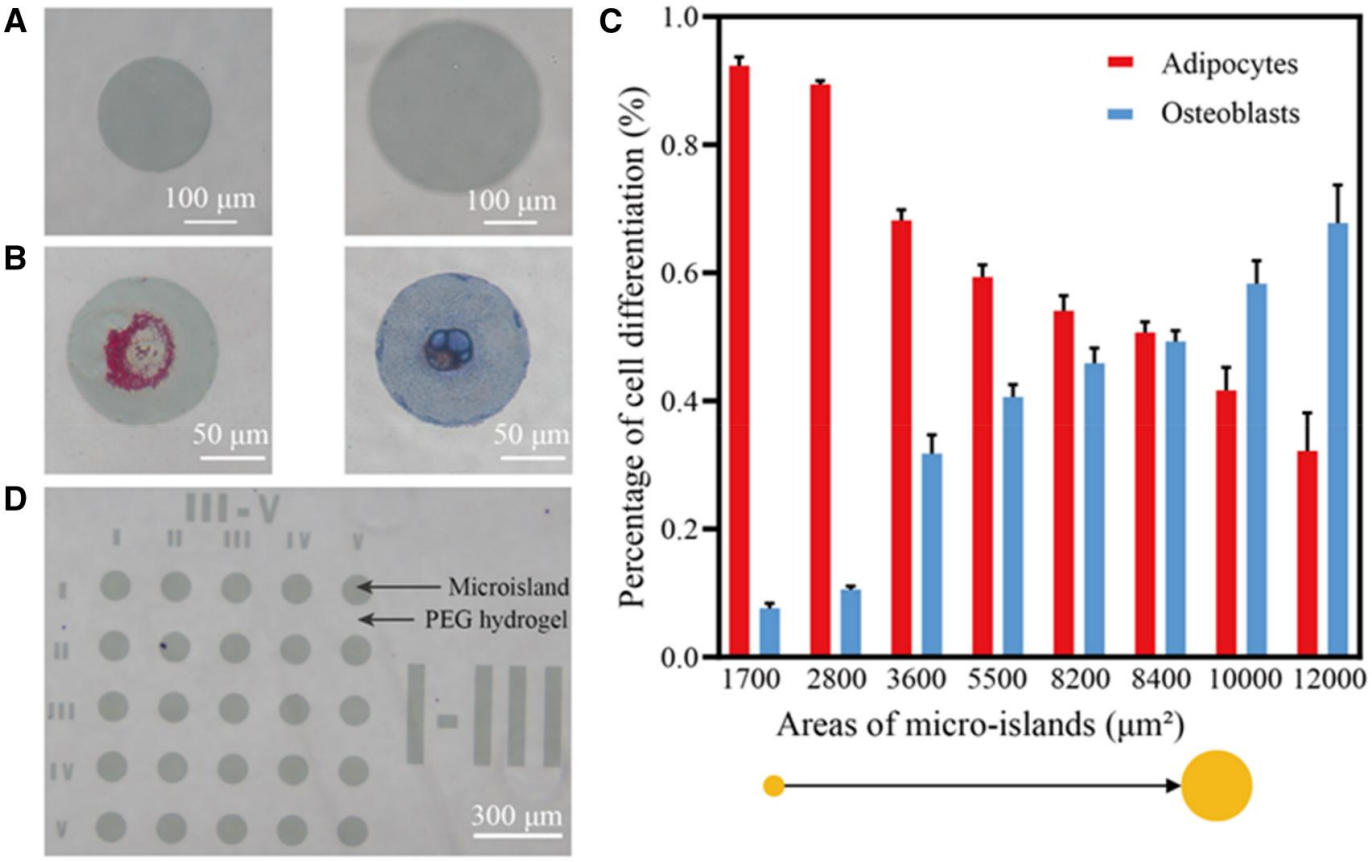
The RGD microarrays on PEG hydrogels for tracing cell differentiation. (**A**) Phase contrast micrographs of RGD microislands with different areas. (**B**) Oil red O staining of lipid droplets for adipocyte and fast blue staining of ALP for osteoblasts differentiated from hMSCs on the RGD microislands. (**C**) Differentiation ratios of hMSCs on RGD microislands of different areas. Data points represent mean ± standard deviation; *n *= 3 hydrogels for each area, and *n *> 100 cells for each hydrogel. (**D**) Phase contrast image of a PEG hydrogel with microislands of 8400 μm^2^ and the markers.

We employed the material surface micropatterning to track the differentiation fate of each individual cell. The prepared micropatterns were characterized with phase contrast photographs (Figure 2A). The areas of the cell-adhesive microislands were 1700, 2800, 3600, 5500, 8200, 8400, 10 000 or 12 000 μm^2^. The differentiation ability of hMSCs on circular RGD microislands was examined. After 2 weeks of induction, hMSCs differentiated into adipocytes or osteoblasts on RGD microislands depending on sizes of cell-adhesive microislands (Figure 2B). We found that the hMSCs on large microislands intended to differentiate into osteoblasts, while more adipocytes appeared on small microislands (Figure 2C), which is consistent with previous reports [9, 10, 51]. Osteogenic and adipogenic differentiation were unbiased with the middle-sized microisland (8400 μm^2^), which was finally selected to culture hMSCs and induce cell differentiation. We designed a microarray with specific labels to facilitate the tracking of hMSCs differentiation at the single-cell level (Figure 2D).

**Figure 3.**
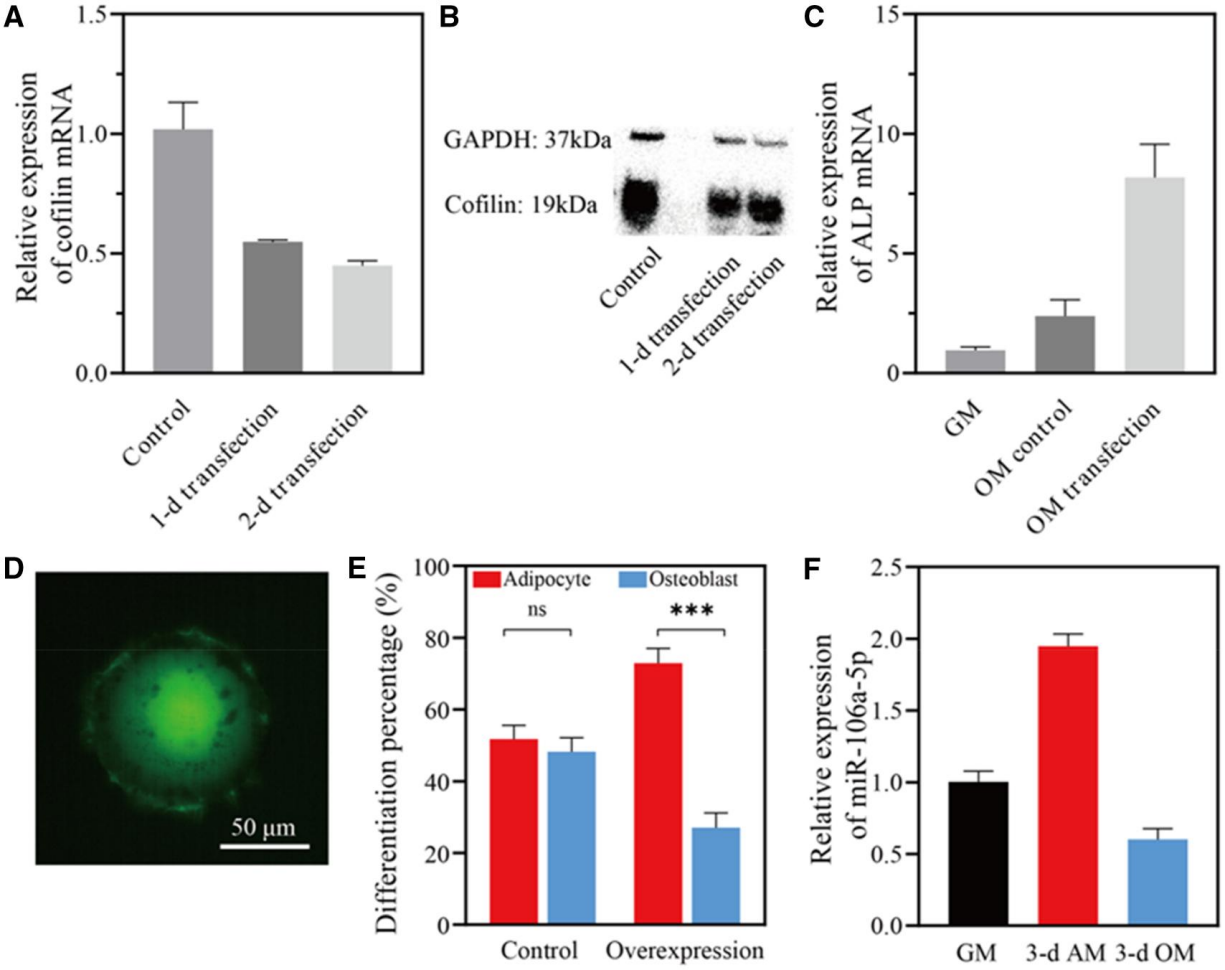
Validation of the target gene and trigger miRNA. (**A**) Relative expression of cofilin mRNA after ssASO Yc transfection. (**B**) Western blot analysis of cofilin after ssASO Yc transfection. (**C**) Relative expression of ALP mRNA of undifferentiated hMSCs in the ground medium (GM), one-week OM induced control group (OM control) and Yc transfected hMSCs (OM transfection). (**D**) The cofilin-overexpressing cell with GFP signal on the RGD microisland. (**E**) Cofilin overexpression and differentiation results. Data points represent mean ± standard deviation; *n *= 178 cells. The *t* test was used for statistical significance analysis between two groups. The statistical significance was symbolized by ‘ns’ (*P *> 0.05), ‘*’ (*P *< 0.05), ‘**’ (*P *< 0.01), ‘***’ (*P *< 0.001)). (**F**) Relative expression of miR-106a-5p after 3-d sole induction. Data are shown in the way of mean ± standard deviation; *n *= 2 wells for qPCR experiments.

Previous research has demonstrated that the differentiation of hMSCs was influenced by a range of signaling pathways [52, 53], with cofilin being a key factor in this process [31–33]. To verify this, we first designed an ssASO (Yc, Supplementary Table S1) that specifically targets cofilin [54, 55]. We found that, one day or two days after transfection, the expression of cofilin in the mRNA level was reduced by roughly half (Figure 3A). The Western blot analysis further showed that Yc significantly inhibited the expression of cofilin protein (Figure 3B). After a week of induction, the cells transfected with Yc expressed a significantly higher mRNA of the osteogenic marker ALP than the control group (Figure 3C), which was in accordance with the prior study [31].

Next, we evaluated a direct correlation between overexpression of cofilin and cell differentiation using the micropatterning platform. Cells with overexpression of cofilin exhibited green fluorescence (Figure 3D), which was marked with specific labels via the micropattern (Figure 2D). We found that cells with cofilin overexpression demonstrated an increased adipogenic differentiation ratio by approximately 3-fold (Figure 3E). In contrast, cells without cofilin overexpression maintained a similar differentiation ratio (Figure 3E). These results indicated that cofilin played an important regulatory role in the osteogenic differentiation of hMSCs, consistently with previous reports [31–33]. As such, cofilin was selected as the target gene for ASO intervention.

Then we examined the expression of miR-106a-5p in hMSCs, which serves as the trigger for the phenotype-specific activation of ASO. Our results demonstrated that after the sole induction for 3 days, the expression of miR-106a-5p in adipogenic-induced cells was considerably higher than that in osteogenic-induced cells (Figure 3F), in line with the results of other studies [34–36]. Taking together, these findings demonstrated that the ASO was selected based on inhibition to cofilin, and miR-106a-5p could serve as a trigger to activate the Y-DNA.

### Design and characterization of Y-DNA targeting cofilin

The Y-DNA structure is composed of three partially complementary ssDNA strands (Supplementary Table S1), with its working principle illustrated in Figure 1B. At the 5' end of Ya, a FAM fluorophore is modified, while the complementary part in Yc carries the quenching group BHQ1 [56, 57]. Conversely, the 5' end of Yc is modified with the CY5 fluorophore, which is paired with the quenching group BHQ2 in Yb [56, 58]. Ya is designed to be complementary to the target miR-106a-5p starting from its 3' end, and specifically, carrying a toehold region for miRNA recognition. As a result, when Ya encounters miR-106a-5p, the Y-DNA structure undergoes partially disassembly, leading to the production of a FAM signal. Simultaneously, the disassembly of Y-DNA exposes a toehold region of Yc that is complementary to mRNA of cofilin. Upon encountering the cofilin mRNA segment, it undergoes hybridization and release of the CY5 signal. Serving as a bridge within this structure, Yb connects Ya and Yc, forming a stable whole.

**Figure 4.**
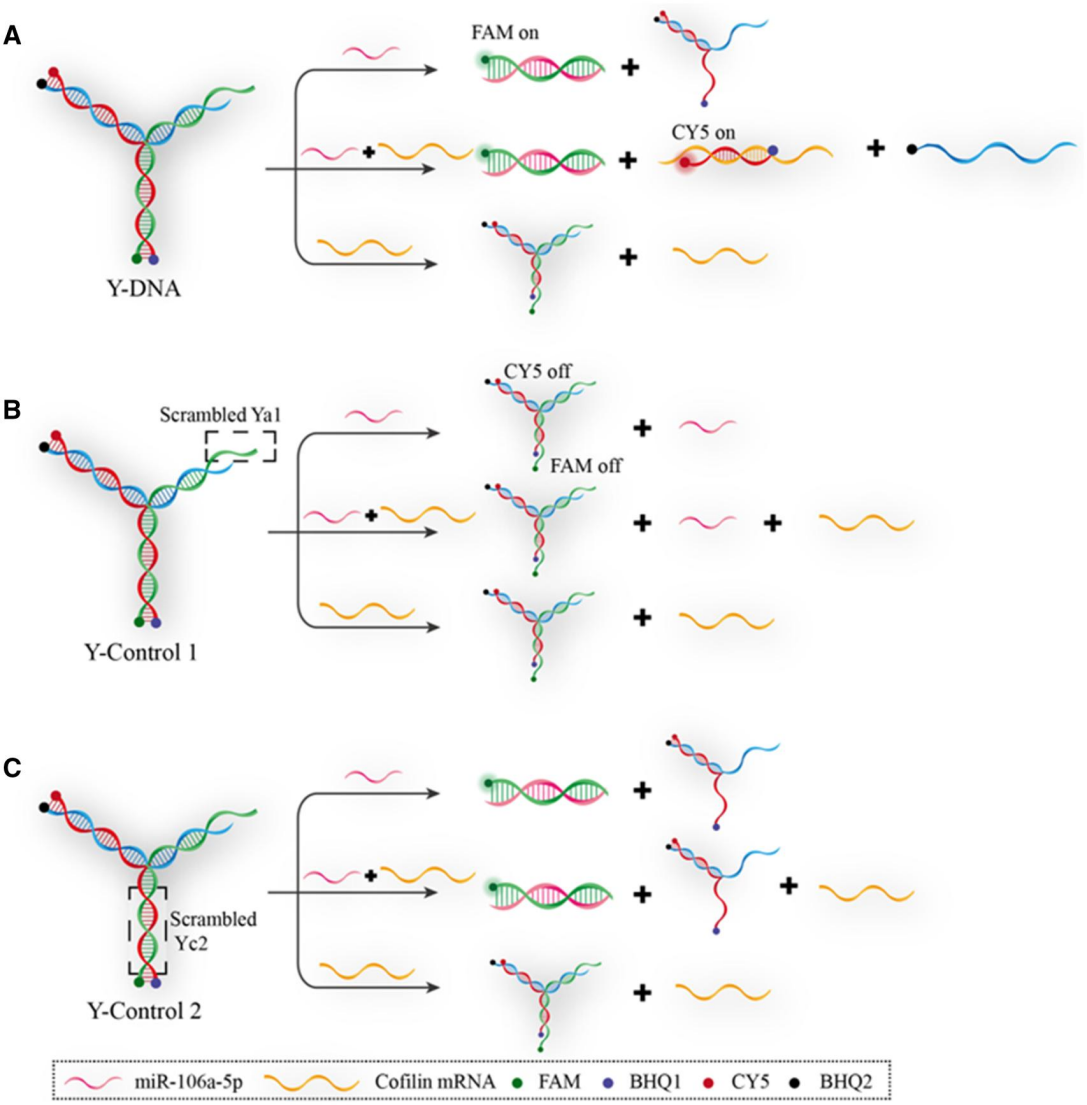
Schemes of the two control Y-DNAs designed to confirm the dynamic properties of the experimental Y-DNA. (**A**) Y-DNA and the disassembly procedures after addition of miR-106a-5p and cofilin mRNA. (**B**) Y-Control 1 contains a scrambled Ya1 strand, preventing disassembly by miR-106a-5p and maintaining structural integrity, with the ASO strand (Yc) identical to that in Y-DNA. (**C**) Y-Control 2 includes a scrambled Yc2 strand, while Ya2 retains the function of Ya, allowing recognition of miR-106a-5p and disassembly. Disassembly of Y-Control 2 by miR-106a-5p releases Yc2, which does not hybridize with cofilin mRNA, leaving its expression unaffected.

To confirm the self-assembly and disassembly of the Y-DNA as a dynamic nanomaterial, we designed two control sets of Y-shaped DNA nanostructures with different scrambled DNA strands. These DNA nanostructures, named as Y-Control 1 and Y-Control 2, possess the same modification of FAM, CY5 and corresponding quenching groups as Y-DNA (Figure 4). Compared to Y-DNA, Y-Control 1 contains a scrambled Ya1 strand, which prevents its disassembly by miR-106a-5p and allows it to maintain its structural integrity, and the ASO strand (Yc) remains the same as that in Y-DNA. Conversely, Y-Control 2 consists of a scrambled Yc2 strand and the function of Ya2 is the same as that of Ya. It can recognize miR-106a-5p, and then, cause Y-Control 2 to be disassembled. However, when Y-Control 2 is disassembled by miR-106a-5p, the released scrambled Yc2 will not hybridize with cofilin mRNA, thus, not affecting the expression of cofilin mRNA.

**Figure 5.**
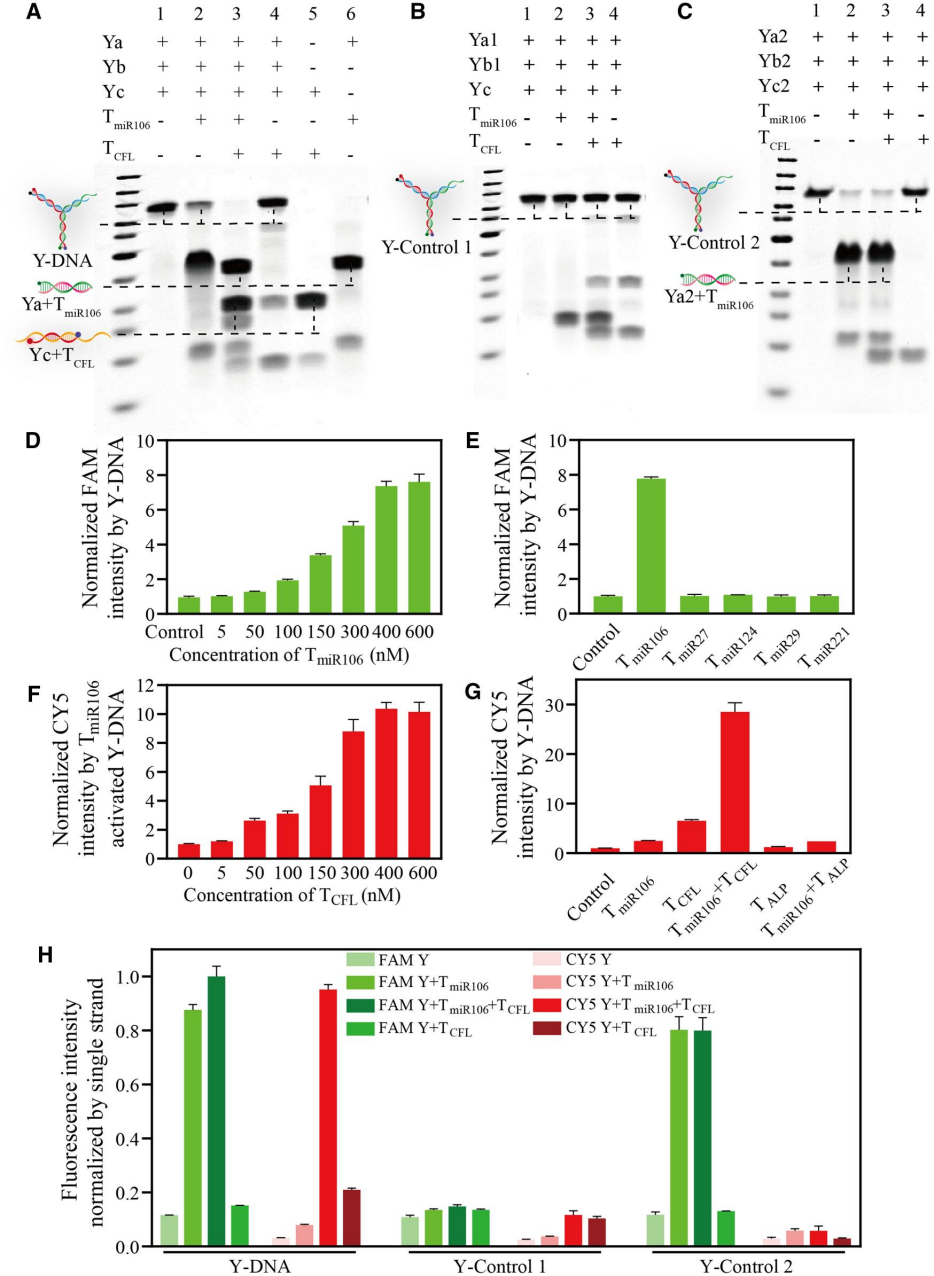
Characterization of experimental Y-DNA and two model Y-shaped DNAs. (**A**) PAGE gel images of Y-DNA confirming the successful formation of Y-DNA and its activation after disassembly triggered by T_miR106_. (**B**) PAGE gel results of Y-Control 1. (**C**) PAGE gel results of Y-Control 2. (**D**) Concentration dependence of T_miR106_ that triggers the disassembly of Y-DNA and produces FAM signal. (**E**) Selectivity test of different miRNA mimics representing the potential interfering factors in cells. (**F**) Concentration dependence of T_CFL_ that hybridizes to Yc and produces CY5 signals. (**G**) Selectivity test of ALP mRNA mimics. (**H**) Fluorescence test results of these three DNA nanostructures under different conditions. Data represent mean ± standard deviation; *n *= 3 for each group.

After the annealing procedure, three ssDNA hybridize to each other to form the Y-DNA. The formation and selectivity of Y-DNA were evaluated using PAGE gel and fluorescence experiments, respectively. Only one band was observed in the results of the PAGE gel (Figure 5A, lane 1), confirming the successful formation of Y-DNA. When only miR-106a-5p mimic (T_miR106_) was introduced, lane 2 showed the presence of a new single heavy band (Ya-T_miR106_ helix, Figure 5A). In contrast, when both T_miR106_ and cofilin mRNA mimic (T_CFL_) were introduced, lane 3 revealed the appearance of two heavy bands (Ya-T_miR106_ and Yc-T_CFL_ helix, Figure 5A). These results align with lane 5 (Yc + T_CFL_) and lane 6 (Ya + T_miR106_) (Figure 5A), indicating that T_miR106_ can trigger the disassembly of Y-DNA. Furthermore, after Y-DNA was disassembled in the presence of T_miR106_, the exposed part of Yc could hybridize with T_CFL_ and completely disassemble Y-DNA. Therefore, it is evident that the disassembly of Y-DNA and the release of Yc only occur when T_miR106_ or T_miR106_ and T_CFL_ coexist. Additionally, lane 4 demonstrates that with the sole absence of T_miR106_, only a small amount of Y-DNA can react with T_CFL_ (Figure 5A), suggesting that Y-DNA can protect cells from the automatic inhibition of cofilin mRNA caused by the releasing of Yc strand.

The PAGE gel results demonstrated that lane 1 exhibited only one band, indicating the successful formation of the two control Y-shaped DNAs (Figure 5B and C). T_miR106_ and T_CFL_ did not hybridize with Y-Control 1 (Figure 5B, lanes 2 and 3), whereas T_miR106_ exclusively disassembled Y-Control 2 (Figure 5C, lane 2). The released Yc2 failed to hybridize with T_CFL_ (Figure 5C, lane 3).

The fluorescence results showed that the signals of FAM and CY5 in intact Y-DNA were extremely low (Figure 5D, E, G and H), confirming the correct formation of Y-DNA. Following the reaction between T_miR106_ and Y-DNA, the signal of FAM gradually increased with the concentration of T_miR106_ (Figure 5D). Meanwhile, the intensity of FAM was not altered by the addition of other miRNA mimics that are typically expressed in hMSCs (Figure 5E), such as T_miR27_, T_miR124_, T_miR29_ and T_miR221_ [59–62], indicating the high specificity of Y-DNA for miR-106a-5p. Once Y-DNA was completely disassembled by T_miR106_, the signal of CY5 was enhanced by increased concentration of T_CFL_ (Figure 5F), suggesting that the release of Yc (ASO) was dependent on the concentration of T_CFL_. The addition of ALP mRNA mimics, which represents the gene widely expressed in MSCs [63], did not affect the CY5 signal (Figure 5G). These results collectively demonstrated that the Y-DNA nanostructure exhibits concentration dependence and high selectivity. In the solution of Y-Control 1, neither T_miR106_ nor T_miR106_ and T_CFL_ together induced significant changes in the FAM and CY5 signals (Figure 5H). In the solution of Y-Control 2, only T_miR106_ hybridized with strand Ya2, leading to a significant amplification of the FAM signal, while T_CFL_ did not elicit a significant change in the CY5 signal (Figure 5H).

### Cellular response of the designed Y-DNA

**Figure 6.**
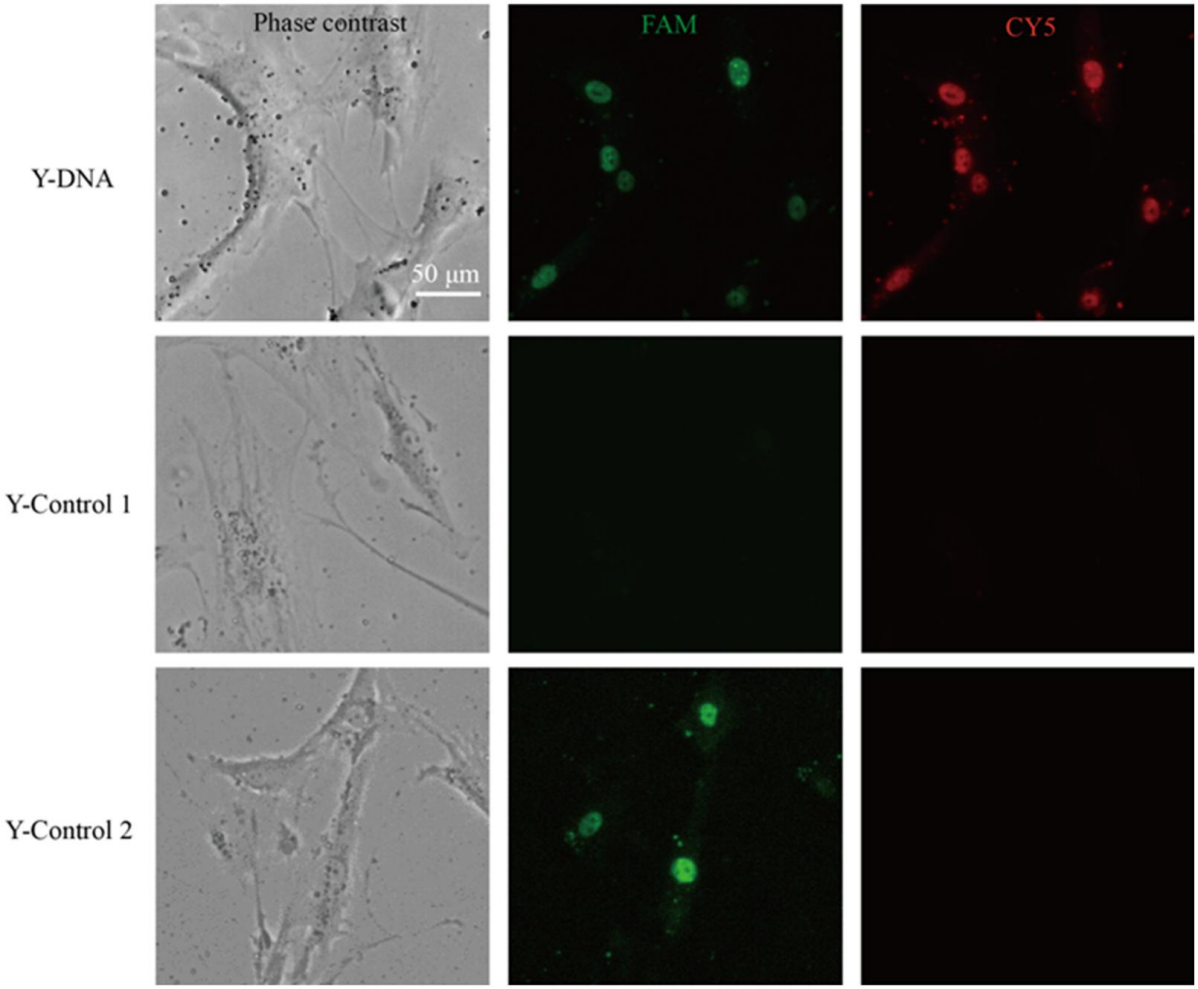
Application of Y-shaped DNA nanomaterials in undifferentiated hMSCs. Phase contrast and fluorescence images for experimental Y-DNA, the two model Y-shaped DNAs as controls. All images have the same scale bar.

After transfection in live and undifferentiated hMSCs, significant FAM and CY5 fluorescence were observed in hMSCs transfected with Y-DNA (Figure 6). In contrast, cells transfected with Y-Control 1 did not exhibit these two fluorescence signals, and cells transfected with Y-Control 2 as another control merely displayed FAM fluorescence (Figure 6). Collectively, these findings indicated that only the experimental Y-DNA specifically hybridized with miR-106a-5p and cofilin mRNA, demonstrating its phenotype-specific activation. In addition, ASOs can function in both the nucleus and the cytoplasm [64, 65]. In our results, we found that FAM and CY5 signals were mostly observed in the nucleus (Figure 6). Therefore, the Y-DNA should primarily exert its effects in the nucleus.

**Figure 7.**
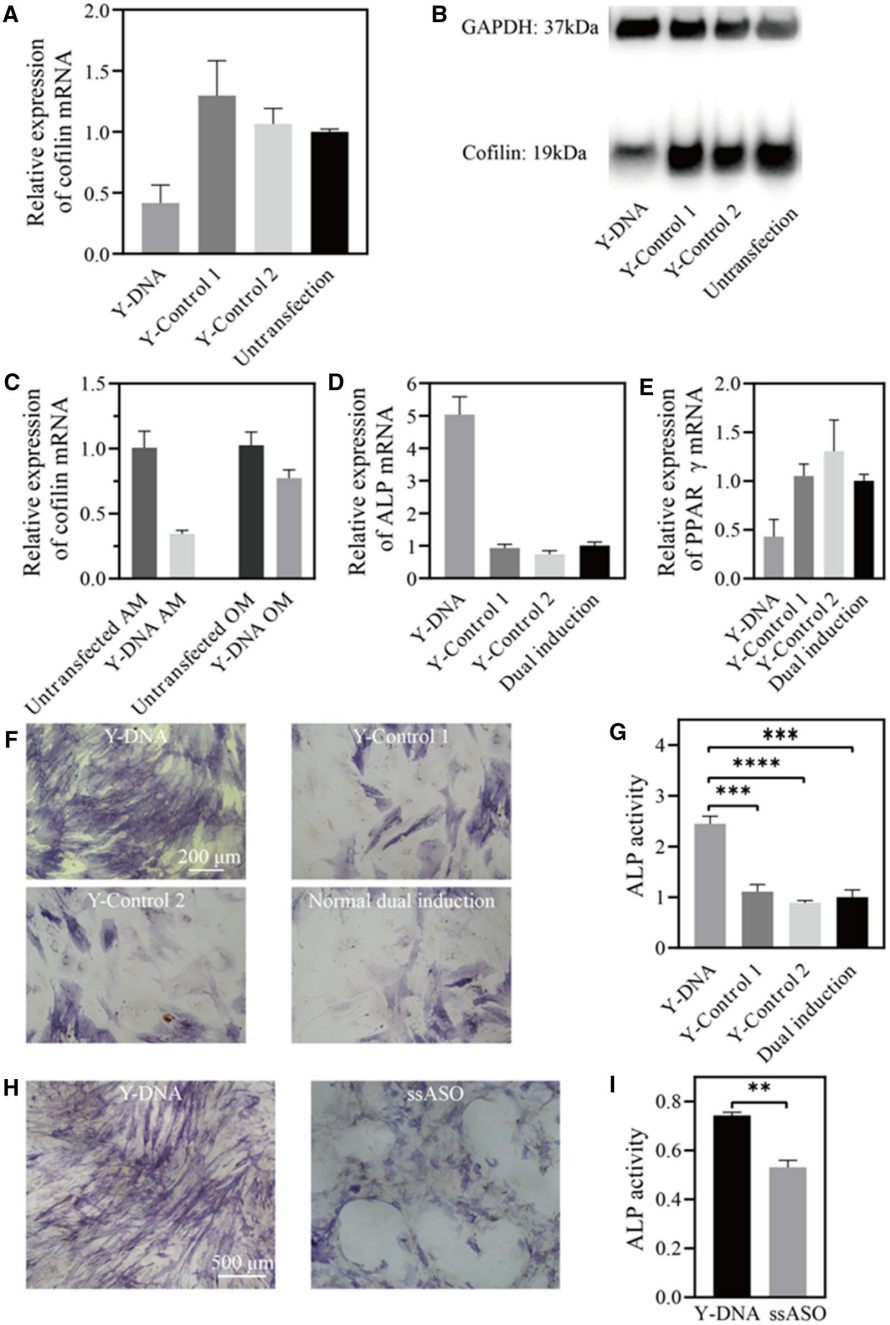
Cell experimental performance of Y-DNA. (**A**) Relative expression of cofilin mRNA from qPCR and (**B**) Western blot analysis of cofilin protein after transfection by different DNA nanostructures in undifferentiated hMSCs. (**C**) Relative expression of cofilin mRNA after Y-DNA transfection in 3-d AM and OM induction hMSCs, respectively. (**D, E**) Relative expression of differentiation markers (ALP, PPARγ) after 7-d dual induction with different DNA nanostructures transfected. (**F**) Bright-field optical micrographs of cells. Oil red O staining for lipid droplets, fast blue staining for ALP results of different Y-DNAs transfected after 7-d dual induction. All images have the same scale bar. (**G**) ALP activity quantitated by optical density at 570 nm and normalized by the mean of Y-Control 1 and Y-Control 2. (**H**) Representative fast blue staining images of 3-d dual induced cells transfected with Y-DNA and ssASO after 7-d dual induction. All images have the same scale bar. (**I**) ALP activity quantitated using optical absorbance at 570 nm. Data points represent mean ± standard deviation; *n *= 2 wells for qPCR experiments, *n *= 3 wells for ALP activity measurement. The *t* test was used for statistical significance analysis between two groups, ‘**’ (*P* < 0.01), ‘***’ (*P* < 0.001), ‘****’ (*P* < 0.0001).

To assess the impact of Y-shaped nanostructures on hMSCs, we next examined the inhibitory effect of three different Y-shaped DNAs on the mRNA level of cofilin. The qPCR results showed that only the cell group transfected with the experimental Y-DNA exhibited a significant reduction in cofilin mRNA expression by more than half (Figure 7A). Additionally, the Western blot results demonstrated that only the Y-DNA decreased the expression of cofilin at protein level (Figure 7B). More importantly, we found much enhanced inhibitory effect of the Y-DNA on cofilin mRNA in adipogenic-induced cells (Figure 7C). It has been demonstrated that high expression of miR-106a-5p is associated with early adipogenic differentiation of hMSCs [33–35]. These findings indicated that Y-DNA could effectively silence the cofilin gene specifically for cells with high expression of miR-106a-5p, confirming the specificity and effectiveness of Y-DNA. Moreover, compared to ssASO Yc, Y-DNA has minimal impact on cell viability (Supplementary Figure S1).

Next, we investigated the enhancement of osteogenic induction by the Y-DNA nanomaterials. Cells were transfected with dual induction medium for three days with three types of DNA (one experimental Y-DNA and two model Y-shaped DNAs as controls), followed by continued dual induction for one week prior to analysis. Comparing the cells treated with model DNAs and cells without addition of any transfected vector, the experimental Y-DNA significantly enhanced the expression of the osteogenic marker ALP (>5 times) (Figure 7D) and inhibited the expression of the adipogenic marker PPARγ (<50%, Figure 7E). Furthermore, the results of terminal differentiation indicated that cells transfected with Y-DNA exhibited a significant increase in ALP expression by about 2.5 times, while the control groups with either Y-Control 1 or Y-Control 2 did not show any significant change (Figure 7F and G). Remarkably, in comparison to ssASO Yc, the experimental Y-DNA significantly enhanced osteogenic differentiation (Figure 7H and I) because of its selective activation and low toxicity. These findings demonstrated that in highly heterogeneous hMSCs, our well-designed Y-DNA, which releases ASO triggered by specific miR-106a-5p, can effectively regulate cell differentiation.

## Discussion

### Fabrication of micropatterns and differentiation of hMSCs

Stem cell differentiation is one of central academic topics in regenerative medicine [66–69]. Beyond soluble factors, extracellular matrix and material cues influence cell behaviors [70–80]. Studies have indicated that cell geometry acts as a driver of phenotype translation of cell phenotype or lineage commitment of stem cells such as osteogenic and adipogenic differentiation [9, 10, 51, 81]. Studies have shown that mechanical cues can also induce stem cell differentiation [82, 83]. This underscores the importance of modulating cellular shape in stem cell differentiation research. In this context, micropatterning technology can effectively control cell shape while mitigating the impact of intercellular contacts on cell differentiation.

Cell differentiation typically requires extended cultivation periods. Classic nonadhesive areas on micropatterned material surfaces may degrade over time, causing the patterns to lose their regulatory function [84]. To address this challenge, Ding's group developed a micropatterning technique based on a strongly fouling-resistant background, generating cell-adhesive peptide RGD patterns on PEG hydrogels for studying stem cell differentiation [85]. Using this technique, we prepared a series of surface patterns with various sizes featuring an antifouling adhesive background. Our investigation into the differentiation behavior of stem cells revealed a close correlation between cell differentiation and cell size, with larger sizes favoring osteogenesis and smaller sizes favoring adipogenesis, consistent with literature reports [9]. As a result, a microisland size (8400 μm^2^) that did not influence cell differentiation significantly was identified and selected for subsequent research.

### The role of the Y-shaped DNA structure

Y-shaped DNA is composed of three single-stranded DNA strands that hybridize with each other. Upon mixing and annealing the three strands, our PAGE gel analysis showed only a single band (Figure 5A, band 1), confirming the formation of the Y-shaped DNA structure. We also employed Nupack online tool (www.nupack.org) to simulate the annealing of three strands, namely Ya, Yb and Yc. The result indicates that these three strands form a Y-shaped DNA structure upon annealing, as depicted in Supplementary Figure S2. The unique trimeric structure of Y-shaped DNA endows it with high thermal and chemical stability, enabling it to remain relatively intact in complex biological environments and resist denaturation or degradation. This stability provides a more reliable basis for applications in biological detection and gene regulation. Y-shaped DNA can be designed to interact with specific DNA sequences, thereby modulating gene expression levels and subsequently influencing cellular processes such as proliferation and apoptosis [86, 87]. In contrast, other geometric structures, such as the tetrahedral DNA, although programmable to some extent, may be less flexible and multifunctional compared with Y-shaped DNA [88].

Conversely, this Y-shaped DNA nanostructure also benefits the delivery efficiency. Studies have shown that DNA nanostructures can significantly improve the stability and delivery efficiency of nucleic acids by forming stable complexes and avoiding lysosomal degradation [89]. Conventional ASOs are less efficient in delivery compared to Y-DNA. This is because DNA nanostructures like Y-DNA can protect nucleic acids from degradation in lysosomes, facilitate their release into the cytoplasm, and enhance cellular uptake through specific targeting mechanisms. Additionally, the programmable nature of DNA nanostructures allows for the design of carriers that can specifically target cells or tissues, further enhancing their delivery efficiency.

### Phenotype-specific regulation of stem cell differentiation

We employed surface micropatterning technology to validate the pivotal regulatory role of cofilin in the differentiation of hMSCs. Following this validation, we explored methods for the specific modulation of this protein. Traditional gene regulation methods, such as CRISPR and siRNA, lack the precision to distinguish between cell types, leading to variable therapeutic outcomes across different cell populations [12, 14]. Our designed Y-DNA nanostructure leverages the specific recognition and response to miR-106a-5p, combined with established ASO technology [21], to ensure that the ASO which targets cofilin, the key regulator of hMSC differentiation, is released exclusively within the specific cellular phenotype of hMSCs. This approach minimizes the accumulation of ASO in non-target cells, thereby enhancing the precision of therapy and reducing off-target effects. Specifically, the design suppresses the expression of cofilin, promoting the differentiation of cells towards osteoblasts rather than adipocytes, which holds significant implications for bone tissue engineering and regenerative medicine. Furthermore, this targeted activation also contributes to reducing potential toxicity risks and is crucial for improving the biocompatibility and safety of the nanostructure, thereby laying the groundwork for clinical applications.

## Conclusion

We have reported a well-designed Y-shaped DNA nanomaterial that facilitates miRNA-triggered release of ASO for cofilin mediated gene silencing and hMSC differentiation regulation. Material surface micropatterning is used to establish a direct correlation between cofilin overexpression and cell fate. Upon transfection into cells by the Y-DNA, the released ASO effectively inhibits the expression of cofilin mRNA and protein, down regulating adipogenic differentiation of hMSCs. The introduction of the Y-DNA leads to a significant enhancement in the osteogenic differentiation of hMSCs in the dual-induction medium towards to adipogenesis and osteogenesis. Based on the dynamic and selective activation, the Y-DNA with designed sequence exhibits greater efficacy with lower toxicity compared to ssASO. These findings highlight the potential of the Y-shaped DNA design strategy, which holds great potential for regulation of stem cell differentiation and regenerative medicine on the gene level.

## Supplementary Material

rbaf043_Supplementary_Data
