# Supplementary material for: Y-shaped DNA as a dynamic self-assembly nanomaterial for phenotype-specific regulation of stem cell differentiation on the gene level
Source: Regen Biomater. 2025 May 14;12:rbaf043. doi: 10.1093/rb/rbaf043 (PMC12202102; doi:10.1093/rb/rbaf043)
Supplement: rbaf043_Supplementary_Data [file rbaf043_supplementary_data.docx]

Supporting Information

**Y-shaped DNA as a dynamic self-assembly nanomaterial for phenotype-specific regulation of stem cell differentiation on the gene level**

Wengang Liu^1^, Ruili Liu^2^, Lok Ting Chu^3^, Xinlei Wang^2^, Jianpeng Wu^1^, Jiandong Ding^2*^ and Ting Hsuan Chen^1, 4, 5*^

^1^Department of Biomedical Engineering, City University of Hong Kong, Hong Kong Special Administrative Region 999077, China

^2^State Key Laboratory of Molecular Engineering of Polymers, Department of Macromolecular Science, Fudan University, Shanghai 200438, China

^3^Department of Biochemistry and Molecular Biology, Guang Dong Medical University, Zhanjiang 524023, China

^4^City University of Hong Kong Shenzhen Research Institute, Shenzhen 518057, P.R. China

^5^Hong Kong Centre for Cerebro-cardiovascular Health Engineering, Hong Kong Science Park, Hong Kong Special Administrative Region 999077, China

*Correspondence authors.

**Jiandong Ding**, Email: [jdding1@fudan.edu.cn](mailto:jdding1@fudan.edu.cn)

**Ting-Hsuan Chen**, Email: [thchen@cityu.edu.hk](mailto:thchen@cityu.edu.hk)

**Table S1. Sequences of oligonucleotides**

| Name | Strand | Sequence (5'--3') |
| --- | --- | --- |
| Y-DNA | Ya | (FAM)G*G*A*A*G*G*T*G*T*T*C*A*A*T*T*C*T*A*C*C*T*G*C*A*C*T*G*T*A*A*G*C*A*C*T*T*T*T |
|  | Yb | C*T*T*A*C*A*G*T*G*C*A*G*G*T*A*G*T*C*G*A*C*A*T*G*A*A*A*G*G*G(BHQ2) |
|  | Yc | (CY5)C*C*C*T*T*T*C*A*T*G*T*C*G*T*T*G*A*A*C*A*C*C*T*T*C*C (BHQ1) |
| Y-Control 1 | Ya1 | (FAM)G*G*A*A*G*G*T*G*T*T*C*A*A*T*T*A*C*G*T*T*C*A*T*G*A*C*C*A*T*C*T*A*T*T*G*C*C*T |
|  | Yb1 | A*G*A*T*G*G*T*C*A*T*G*A*A*C*G*T*T*C*G*A*C*A*T*G*A*A*A*G*G*G(BHQ2) |
|  | Yc | (CY5)C*C*C*T*T*T*C*A*T*G*T*C*G*T*T*G*A*A*C*A*C*C*T*T*C*C (BHQ1) |
| Y-Control 2 | Ya2 | (FAM)G*A*G*T*A*G*A*A*G*G*C*G*T*T*C*T*A*C*C*T*G*C*A*C*T*G*T*A*A*G*C*A*C*T*T*T*T |
|  | Yb2 | C*T*T*A*C*A*G*T*G*C*A*G*G*T*A*G*T*A*A*T*A*C*A*A*T*C*T*G*G*C*C(BHQ2) |
|  | Yc2 | (CY5)G*G*C*C*A*G*A*T*T*G*T*A*T*T*C*G*C*C*T*T*C*T*A*C*T*C(BHQ1) |
| T_miR106_ | Mimic of miR-106a-5p | AAAAGTGCTTACAGTGCAGGTAG |
| T_miR27_ | Mimic of miR-27a-3p | TTCACAGTGGCTAAGTTCCGC |
| T_miR124_ | Mimic of miR-124-3p | TAAGGCACGCGGTGAATGCCAA |
| T_miR29_ | Mimic of miR-29a-5p | ACTGATTTCTTTTGGTGTTCAG |
| T_miR221_ | Mimic of miR-221-5p | ACCTGGCATACAATGTAGATTT |
| T_CFL_ | Mimic of cofilin mRNA | AAGGTGTTCAACGACATGAAA |
| T_ALP_ | Mimic of ALP mRNA | ACGTGGCTAAGAATGTCATC |

Note: “*” means phosphorothioate modification.

**Table S2. Primer Sequences of Relevant for qPCR**

| Name | Strand | Sequence (5'--3') |
| --- | --- | --- |
| GAPDH | Forward | GGATTTGGTCGTATTGGG |
|  | Reverse | GGAAGATGGTGATGGGATT |
| ALP | Forward | ACGTGGCTAAGAATGTCATC |
|  | Reverse | CTGGTAGGCGATGTCCTTA |
| PPARγ | Forward | GACCACTCCCACTCCTTTGA |
|  | Reverse | CGACATTCAATTGCCATGAG |
| Cofilin | Forward | GATAAGGACTGCCGCTATGC |
|  | Reverse | GCTTGATCCCTGTCAGCTTC |


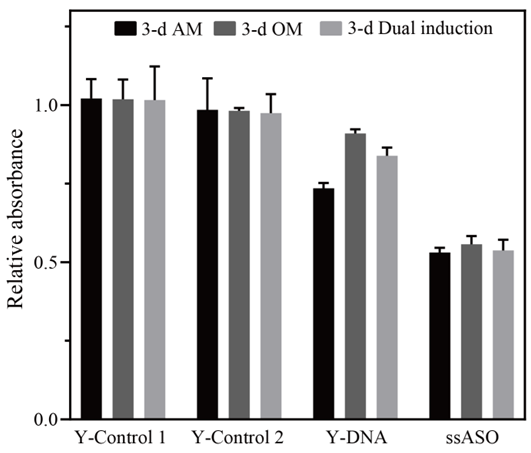


Figure S1. Cell viabilities of hMSCs after transfected with different DNAs. The stem cells were induced in adipogenic medium (AM), osteogenic medium (OM) or mixture medium (dual induction). Optical density (OD) values are normalized by the mean of the two control Y-shape DNAs. For each group, *n* = 3.


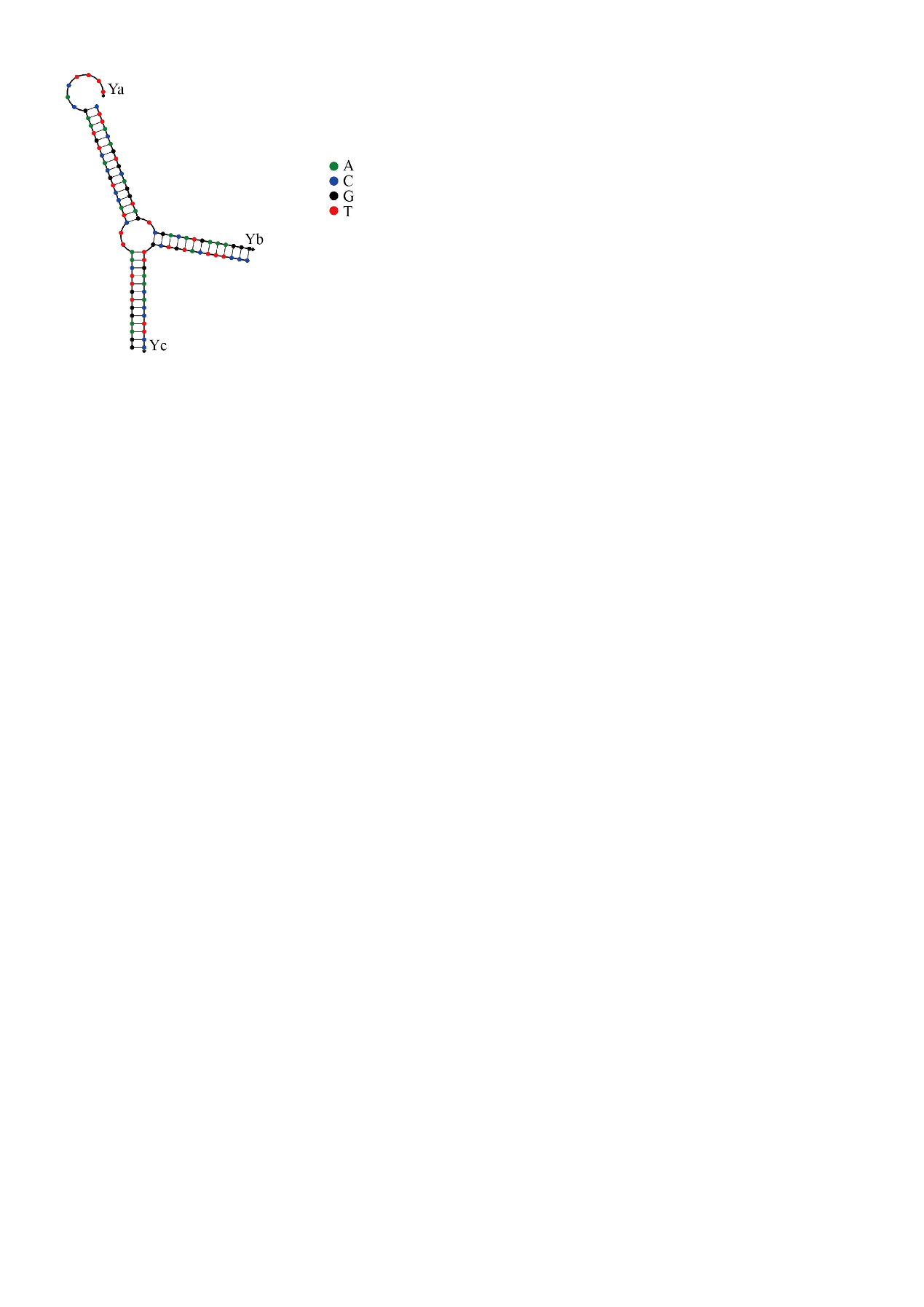


Figure S2. The simulation result of Y-shaped DNA structure using Nupack.
